# Cheek alveolar soft part sarcoma recurrence at the primary site during follow-up: a case report and review of the literature

**DOI:** 10.1186/s12903-024-04431-2

**Published:** 2024-06-13

**Authors:** Wenyu An, Zhongxu Xue, Huifang Zhuo, Ning Wang, Lian Meng, Wei Jia

**Affiliations:** Department of Pathology and Key Laboratory for Xinjiang Endemic and Ethnic Diseases, Shihezi University School of Medicine/The First Affiliated Hospital, Shihezi University, Shihezi, China

**Keywords:** Alveolar soft part sarcoma(ASPS), Cheek, Tumor recurrence at the primary site, Fluorescence in situ hybridization (FISH), Immune checkpoint inhibitors (ICPis) - associated diabetes

## Abstract

**Background:**

Alveolar soft part sarcoma (ASPS) occurs most often in the deep muscles or fascia of the extremities in adults, with only 3.4% of these tumours originating from the head, face and neck. To date, only 17 cases of buccal ASPS have been reported, including the case presented here. Only one case of ASPS recurrence at the primary site, similar to our case, has been reported thus far. Immune checkpoint inhibitors (ICPis)-associated diabetes, with an estimated incidence of 0.43%, is usually seen in older cancer patients and has not been reported in younger people or in patients with ASPS.

**Case presentation:**

A 24-year-old male patient presented with a slowly progressing right cheek mass with a clinical history of approximately 28 months. Sonographic imaging revealed a hypoechoic mass, which was considered a benign tumour. However, a pathological diagnosis of ASPS was made after excision of the mass. Five days later, functional right cervical lymph node dissection was performed. No other adjuvant therapy was administered after surgery. In a periodic follow-up of the patient six months later, blood-rich tumour growth was noted at the primary site, and Positron emission tomography-computedtomography (PET-CT) ruled out distant metastasis in other areas. The patient was referred to the Ninth People’s Hospital of Shanghai Jiaotong University. Due to the large extent of the mass, the patient received a combination of a Programmed Cell Death Ligand 1(PD-L1) inhibitor and a targeted drug. Unfortunately, the patient developed three episodes of severe diabetic ketoacidosis after the administration of the drugs. A confirmed diagnosis of ICPis-associated diabetes was confirmed. After the second operation, the postoperative pathological diagnosis was ASPS, and the margins were all negative. Therefore, we made a final clinical diagnosis of ASPS recurrence at the primary site. Currently in the follow-up, the patient is alive, has no distant metastases, and undergoes multiple imaging examinations every 3 months for the monitoring of their condition.

**Conclusions:**

In analysing the characteristics of all previously reported cases of buccal ASPS, it was found that the clinical history ranged from 1 to 24 months, with a mean of approximately 3 to 9 months. Tumour recurrence at the primary site has been reported in only one patient with buccal ASPS, and the short-term recurrence in our patient may be related to the extraordinarily long 28-month history. ICPis-associated diabetes may be noted in young patients with rare tumours, and regular insulin level monitoring after use is necessary.

## Background

Alveolar soft part sarcoma (ASPS) is usually described as a nonulcerated, painless, slowly enlarging mass with no clinical specificity, in which ultrasound and computed tomography (CT) also provide some help for preoperative diagnosis. On ultrasound, an ASPS appears as a hypoechoic mass, but ultrasound is of limited help in diagnosis [1]. Currently, the diagnosis of ASPS in our centre mainly depends on pathology. Although the exact histogenesis of ASPS is still unclear, its morphologic, immunohistochemical, electron microscopic, and genetic features are well characterized, which contributes to an accurate histopathological diagnosis. ASPS is characterized by polygonal tumour cells arranged in an alveolar or organ-like pattern, separated by fibrovessels, with a uniform morphology [2]. As malignant tumours of mesenchymal origin, alveolar soft tissues are diffusely and strongly positive (nuclear expression) for the transcription factor E3 (TFE3) gene. One of the most common gene fusions is ASPSCR1::TFE3, and this fusion requires differential diagnosis from translocation-associated renal cell carcinoma (RCC) [3].

## Case presentation

**Fig. 1:**
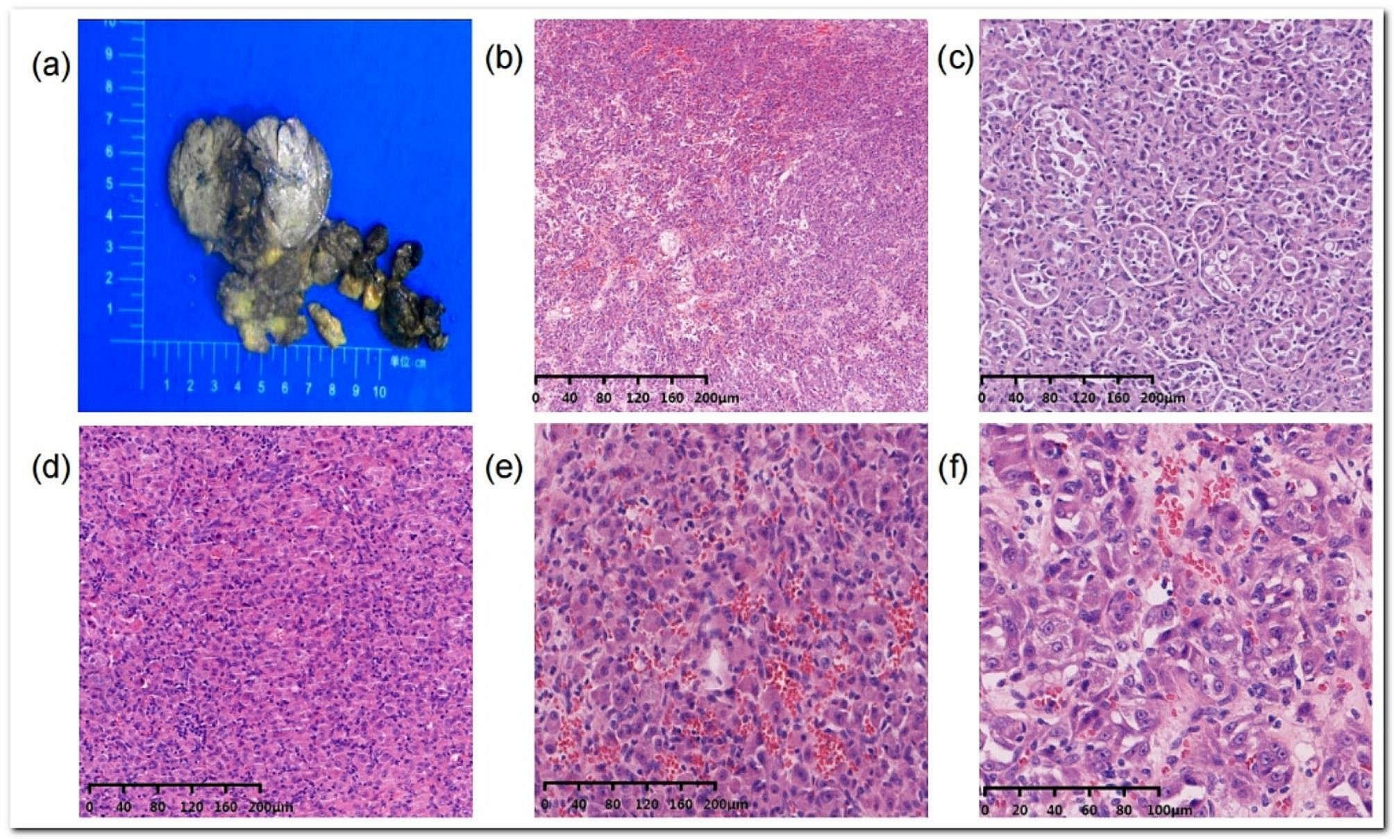
**a**: Resected specimen. **b**: Tumor cells arranged in an alveolar-like pattern. **c**: Tumor cells arranged in solid structures. **d**: Clear demarcation between alveolar-like and solid structures. **e**: rich blood sinuses could be seen in the cell nest **f**: Cellular atypia is significant, with large nuclei and hyperchromatic nuclei

**Fig. 2:**
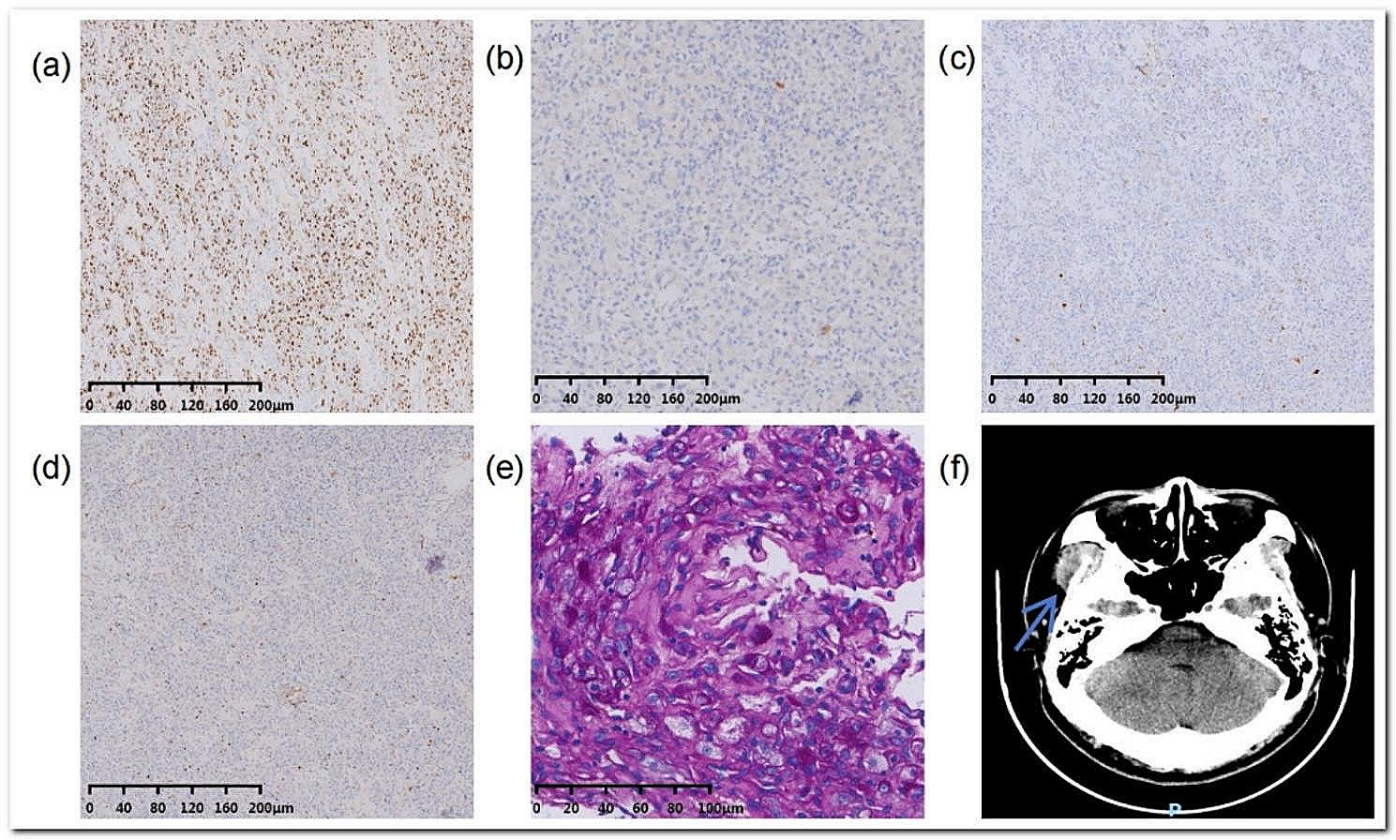
Immunohistochemical staining of primary tumors. **a**: TFE3: diffuse and strong positive cytoplasm of tumor cells. **b**: MyoD1: tumor cells were negative. **c**: Desmin: tumor cells were positive. **d**: Ki67 staining: The Ki67 index hit 8%. **e**: PAS positive shows rod-shaped bodies. **f**: CT-enhanced reconstruction from the arch of the eyebrow to the supraclavicular bone shows irregular soft tissue shadows in the right temporalis muscle region

In June 2021, the patient, 24 years old, was referred to our hospital with the complaint of mild swelling and pain in the cheek, which worsened after eating. The patient had a more than two-year history of a facial mass. Ultrasonography at the former hospital revealed a hypoechoic mass, and a diagnosis of a benign adenogenetic mass was considered likely; therefore, as only local enlargement was observed, the oropharyngeal mass was removed at the First Affiliated Hospital, Shihezi University. However, the pathological diagnosis was buccal ASPS. The mass was approximately 30 mm in size and the cut surface was solid with a reddish yellow colour (Fig. 1a). Histopathologic examination at low magnification revealed an infiltrating lesion, and the tumour cells were arranged in an acinar and solid structure under the microscope and had a unique and characteristic nested or organoid growth pattern (Fig. 1b, c, d). The size and shape of the nests tended to be consistent, but there were also some differences. Under low magnification, the tumour cells were arranged in an “organ-like” or “vesicular” pattern, with a network of slit-like or blood-sinus-like capillaries between the vesicles [4]. Some cells had an empty cytoplasm, some cells had a rich cytoplasm containing eosinophilic red granules, and rich blood sinuses could be seen in the cell nest (Fig. 1e). A few cell nuclei were large and deeply stained, there was atypia, and there was no clear nuclear division (Fig. 1f). However, the cells were disorganized, apolar, and highly malignant, which may be associated with late in situ recurrence [5]. Immunohistochemistry revealed TFE3 (+), MyoD1 (-), Ki67 (8%), Desmin (partial +), S100 (-), SMA (interstitial+), CD56 (weak +), and periodic acid–Schiff (PAS)-positive and amylase-resistant rod-shaped crystals in the cytoplasm of the tumour cells (Fig. 2a-e). Positron emission tomography-computedtomography (PET-CT) revealed increased FDG metabolism in the surgical area and adjacent muscle tissues and multiple enlarged lymph nodes in the right submandibular area. After the pathological results were confirmed, functional right cervical lymph node dissection was performed with the consent of the patient’s family, and no chemotherapy or targeted therapy was administered after the operation.

At the follow-up examination in our hospital six months later, an irregular-density shadow was observed in the soft tissue of the right temporal muscle area on a nonconstrast CT scan, and the enhanced scan showed obvious inhomogeneous enhancement, which was considered to indicate recurrence (Fig. 2f). The mass was larger than before. Moreover, the patient did not have any somatic symptoms. In January 2022, the patient was referred to the Ninth People’s Hospital of Shanghai Jiao Tong University, where it was proposed that he receive a six-month preoperative combination of targeted therapy (apatinib) and an ICPi to treat the large mass. The patient was administered the Programmed Cell Death Ligand 1 (PD-L1) inhibitor and targeted drug combination for two months in January and February and the targeted drug alone in March due to the restrictions for novel coronavirus epidemic prevention and control. Acute diabetic ketoacidosis developed while the patient stayed at home. Three episodes of diabetic ketoacidosis occurred within a short period of time after emergency admission, with a C-peptide value of 0.38 ng/ml and a percentage of glycated serum albumin of 28.3%. After diagnosis, the patient denied a family history of diabetes mellitus, and he was diagnosed with ICPis-associated diabetes mellitus with ketoacidosis. He received a half-month course of insulin treatment. At this point, the patient strongly requested early surgery, and the size of the mass had reduced to approximately 47 mm, similar to the size of the primary mass. Therefore, an extracranial enlarged resection of the right infratemporal fossa mass was performed under general anaesthesia with a prefemoral extracellular skin flap on June 8, 2022. Postoperative pathology revealed postoperative recurrence of the ASPS in the surgical area of the right face with negative margins. The immunohistochemistry results were as follows: TFE3 (+), MyoD1 (a small amount of cytosol +), Ki67 (1%), CD34 (vascular endothelial cells +), Desmin (-), S100 (-), SMA (a small amount of +), and CD56 (-). In the comparison of the immunohistochemistry results before and after recurrence, the Ki67 index decreased from 8 to 1%, possibly due to the weakening of tumour invasion after recurrence, which may be due to the efficacy of preoperative adjuvant chemotherapy. In previous studies on pituitary tumours, the Ki67 index appeared to correlate with the degree of malignant aggression [6]; however, no study has revealed a link between Ki67 and tumour recurrence [7]. However, Ki67 values are reported relatively rarely for our tumour patients; therefore, conclusions cannot be drawn from our results alone, and further research is needed. The results of fluorescence in situ hybridization (FISH) showed that more than 50% of the tumour cells had red and green signal segregation in the nuclei, indicating that the TFE3 gene was damaged, suggesting nonequilibrium translocation of the TFE3 gene. The patient underwent regular maxillofacial CT with contrast and colour ultrasound of the neck and axillary lymph nodes, noncontrast spiral CT of the lungs, and colour ultrasound of the abdomen every three months to rule out recurrence and distant metastasis. To date, the patient has not experienced any in situ recurrence or distant metastasis in regular follow-up with anlotinib hydrochloride. However, the patient lost pancreatic islet function and must receive insulin maintenance therapy for the remainder of his life.

## Discussion and conclusion

### Clinical presentation

The World Health Organization’s classification of soft tissue tumours classifies ASPS as “tumours of indeterminate differentiation”. ASPS occurring in the cheeks are extremely rare, with only 17 cases reported to date, as shown in Table 1 [8–16]. By comparing the clinical manifestations, we found that the mass grew faster than the in situ tumour during recurrence, and the mass was larger in size, but the Ki67 index decreased, which indicated that the tumour was less malignant and invasive, which might be due to the efficacy of our preoperative neoadjuvant chemotherapy or the characteristics of tumour recurrence. The small molecule form of apatinib, the first worldwide targeted antiangiogenic drug, has been proven to be reliable and highly effective in the treatment of advanced gastric cancer, and its efficacy in the treatment of soft-tissue sarcomas has also been demonstrated in previous literature [17]. In previous studies, immunotherapy targeting Programmed Cell Death Ligand 1 (PD-1) and PD-L1 showed good therapeutic effects when administered in combination with other drugs [18]. This combination was the initial treatment we considered, but unfortunately, our patient developed ICPis-associated diabetes with ketoacidosis. According to reports, Immune checkpoint inhibitors (ICPis)-associated diabetes mellitus is a special type of diabetes mellitus [19]. The incidence of ICPi-associated diabetes mellitus is less than 1%, and ICPi-associated diabetes mellitus is typically defined by a prompt onset of hyperglycaemia, a rapid worsening of endogenous insulin deficiency, and a very high incidence of diabetic ketoacidosis, which poses a serious threat to patient safety. The rapid onset of abnormally elevated blood glucose combined with ketoacidosis, no significant increase in glycosylated haemoglobin, and an absence of pancreatic islet β-cell function reflect the severity of this condition and a certain degree of clinical similarity with fulminant type 1 diabetes mellitus (FT1DM) [20]. At present, the classification of ICPi-associated diabetes mellitus is not clear, and patients generally present low levels of insulin and C-peptide. Diabetic islet autoantibodies may be present. In half of patients, GAD antibodies remain positive. The diagnosis of ICPi-associated diabetes mellitus is relatively straightforward, and most patients have a history of taking immunosuppressants due to the onset of malignancy, but it is also necessary to distinguish ICPi-associated diabetes mellitus from FT1DM. Notably, previous reports have focused on elderly people` with cancer, and the mean age of onset was 61.7-years [21]. Whether the severity of our patient’s three severe episodes of diabetic ketoacidosis is inversely correlated with the age of onset is worth exploring. The patient in this case was a young adult with a short duration of diabetes mellitus, which is rare. In addition, 76% of patients with ICPis-associated diabetes mellitus were found to carry the type 1 diabetes susceptibility gene HLA-DR4, and patients diagnosed with a single ICPi-induced autoimmune disease and with high-risk HLA alleles require close monitoring [22]. In terms of treatment, unlike other endocrine-related toxicities, glucocorticoids are not recommended for patients with ICPi-associated diabetes mellitus, and the main treatment is multiple insulin injections and symptomatic support. First and foremost, diabetes is not a contraindication to continued treatment with PD-1 or PD-L1 inhibitors. Immunotherapy should be discontinued immediately only in the acute phase of diabetes, i.e., hyperglycaemic crisis (diabetic ketoacidosis or hyperosmolar syndrome). The initiation of ICPis may be considered for patients transitioning to the chronic phase. Blood glucose should be monitored throughout the course of the disease [23]. Given the obvious variability and uncertainty in the timing of the emergence of adverse effects of such drugs, every clinician should pay close attention to the possible adverse effects of such drugs in patients who are about to use, are using, or have used ICPis; obtain informed consent for the use of these drugs; and perform blood glucose testing and assessment of endocrine gland function on a regular basis, which is conducive to early identification, timely intervention, and follow-up in later stages. Since there are no previous case reports of cheek ASPS recurrence at the primary site and ICPis, we will continue to follow and monitor the long-term prognosis of our patient and look forward to more case reports to deepen our understanding of this disease.

**Table 1:** Clinical characters of alveolar soft part sarcoma cases of the cheek origin

Number	Case	Sex/Age	Maximum dimension (cm)	Location	Tumour duration(month)	Meta/Rec	Treatment	Follow-up (months)	Prognosis	Pathological findings
1	This case	M/24	4.7	Cheek	28	R	S	18	N	A + S
2	2021, Katsutoshi Hirose [8].	F/21	2	Cheek	5	N	S	14	N	A + S
3	2020, Kimi et al. [9]	F/25	1.2	Cheek	NM	N	S	NM	N	A
4		M/22	NM	Buccal Space	NM	N	S + C	22	N	A
5	2014, Wang et al. [10]	F/36	6	Cheek	NM	N	S + R	24	N	A
6	2001, Charrier et al. [11]	F/54	10	Cheek	2	M	C	3	D	A
7	2010, Min et al. [12]	M/37	2.3	Cheek	9	N	S	24	N	S
8	2013, Argyriset al. [13]	M/13	3.2	Buccal Mucosa	2	N	S + C	12	N	S
9	2014, BrandonT.Mullins [14]	F/39	2.1	Parotid gland	24	N	S + R	168	N	A
10	2015, Wang et al. [15]	F/24	1.8	Cheek	NM	N	NM	14	NM	A
11		F/29	2.8	Cheek	NM	N	NM	67	NM	A
12		M/5	5.5	Cheek	NM	R	NM	60	NM	A
13		M/57	4	Cheek	NM	N	NM	60	NM	A
14	2019, Asano et al. [16]	F/11	NM	Cheek	NM	M	NM	NM	NM	NM

S: surgical excision, R: radiotherapy, C: chemotherapy, Rec: recurrence, M: metastasis, N: no evidence of recurrence, NM: not mentioned, D: died, A + S: alveolar growth pattern and solid growth pattern, A: alveolar growth pattern or nests like pattern, S: solid growth pattern

### Histopathology

Pathologically, ASPSs consist of “nests” of cells loosely arranged along the connective tissue, with a loss of intercellular bonding in the centre of the nests. Nests are usually accompanied by necrosis and separated by connective tissue containing thin-walled luminal blood vessels, resulting in the hallmark “alveolar” pattern. A few cases in which tumour cells mutate and grow in solid or sheet-like patterns have also been reported. Fanburg-Smith et al. [24], in their previous report on tongue ASPS, noted that the solid growth pattern predominates in adolescents and children, whereas the alveolar growth pattern is predominantly seen in older individuals. ASPSs need to be differentiated from the following tumours with large cells with nests of eosinophilic/hyaline cytoplasm: (1) ASPSCR 1-TFE 3 translocation in renal cell carcinoma (RCC), (2) adrenocortical carcinoma (ACC), (3) metastatic hepatocellular carcinoma (HCC), (4) paraganglioma, (5) granulosa cell tumour, (6) perivascular epithelioid cell tumours (PEComas), (7) malignant melanoma, and (8) adenovesicular rhabdomyosarcoma. All these differential diagnoses can be ruled out by appropriate immunohistochemical labelling. ASPSs are negative for epithelial, melanocytic and neuroendocrine markers. ASPSs can be positive for boldin and NSE, but this is not as well characterized. According to the FISH results, red‒green signal separation was detected in the nuclei of > 50% of the tumour cells in our patient, suggesting TFE3 gene damage; however, the exact type of fusion is still unclear. The ASPSCR 1::TFE 3 fusion protein is the most common type of fusion, and the HNRNPH 3::TFE 3, DVL 2::TFE 3, and PRCC::TFE 3 fusions are the most recently reported other types of fusions [25].

### Recurrence

Commonly, an ASPS usually presents as a nonpainful, slowly growing mass, whereas an orthotopic recurrent ASPS not treated with chemotherapy presents as a noninvasive, fast-growing mass. However, whether such masses exhibit more malignant biological behaviour and a greater probability of distant metastasis is unknown. Previous cases have shown that metastasis is an important adverse prognostic factor, and a patient with a recurrent alveolar soft-tissue sarcoma of the left eye died of brain metastases after 132 months of follow-up. Thus, longer follow-up is needed to determine whether in situ recurrence affects prognosis.

**Table 2:** Clinical characters of recurrence at the primary site alveolar soft part sarcoma cases

Number	Case	Sex/Age	Maximum dimension (cm)	Location	First treatment	Recurrence (month)	Recurrence Maximum dimension(cm)	Recurrence Treatment	Follow-up (month)	Prognosis
1	This case	M/24	4.7	Cheek	S	6	5.8	S + O	18	L
2	2020, Weimin He [27].	F/9	3.5	left eye	S + R	84	1.3	S + R	132	D
3		F/12	2.4	left eye	S	2	4.2	S + R	36	L
4		M/1	3.8	left eye	S	36	4.2	S + R	NOW	L
5	2006, Andrew L [28].	F/25	NM	bladder	S	11	NM	S	11	L
6	2005, Hakan Emmez [29].	F/11	NM	Left frontal	S + R + C	45	NM	S + C	9	L
7	2013, Ahn et al. [30]	F/9	4.6	Cerebell Opontine angle	S + R + C	29	4.6	S + R	NM	L
8	2022, Xiaochuan Wu [31].	F/9	5	left posterior chest	S + C	43	NM	C	NM	NM
9	2005, Kanhere [32].	F/42	3.5	tongue	S	24	NM	S + R	NM	L
10	2015, Hong-Wei Wang [15].	M/11	3	Dorsal tongue	NM	5	NM	NM	15	L
11		F/14	5	Ventraltongue and floor of mouth	NM	5	NM	NM	15	L
12		F/16	3.3	Base of tonguE	NM	19	NM	NM	35	L
13		M/10	3.5	Base of tonguE	NM	NM	NM	NM	77	L
14		F/6	5	Ventraltongue and floor of mouth	NM	6	NM	NM	60	L
15		F/27	5	Gingiva	NM	46	NM	NM	49	L
16	1999, Bentley [33].	F/5	NM	Lingual Left midline	S + R	12	NM	NM	24	NM

S: surgical excision, R: radiotherapy, C: chemotherapy, O: other new therpy way, Rec: recurrence, M: metastasis, N: no evidence of recurrence, NM: not mentioned, D: died, A + S: alveolar growth pattern and solid growth pattern, A: alveolar growth pattern or nests like patter, S: solid growth pattern, L: live, D: died

In the Japanese Musculoskeletal Oncology Group (JMOG) study, patients who underwent marginal resection and RT had no local recurrence, whereas 57% of patients who underwent marginal resection experienced only local recurrence. These data suggest that adjunctive RT in the setting of inadequate surgical margins is clinically beneficial [26]. There are reports of ocular recurrence that suggest a greater probability of short-term recurrence in patients who do not receive postoperative radiotherapy or targeted therapy. As summarized in Table 2, when comparing the time to tumour recurrence at the primary site, postoperative combined adjuvant therapy is helpful for delaying recurrence [15, 27–33]. The current view of adjuvant therapy is controversial: having a slowly progressive clinical course often leads to delayed diagnosis and treatment. By comparing the size of the tumours before and after recurrence, the patients who underwent adjuvant therapy experienced tumour shrinkage from 3.5 cm to 1.3 cm after recurrence, whereas the size of the tumours before and after recurrence remained unchanged in the patients who underwent surgical resection alone or increased in size, as shown in our case. However, the pros and cons of this delay in recurrence on long-term prognosis are not known. It has also been shown that the ASPSs of elderly patients with positive margins and a long time to first presentation do not recur, which may suggest tumour inertia. However, our patient was found to have a mass for 28 months at the time of consultation, which is the longest reported time to first recurrence in the buccal region; this may be one of the reasons for his short-term orthotopic recurrence, and early detection and eradication of the tumour is still the most important means of treatment at present. Since both our patient and a six-year-old boy were found to experience in situ recurrence at 6 months postoperatively, it may be more beneficial for the patient to appropriately shorten the follow-up interval, and we recommend follow-up visits at 1, 2, 4 and 6 months postoperatively [15].

## Data Availability

Data on patient and case details are available from the author on reasonable request.

## Abbreviations

ASPS: Alveolar soft part sarcoma
ICPis: Immune checkpoint inhibitors
PET-CT: Positron emission tomography-computedtomography
PD-L1: Programmed Cell Death Ligand 1
FISH: Fluorescence in situ hybridization
TFE3: The transcription factor E3
RCC: Renal cell carcinoma
CT: Computed tomography
PD-1: Programmed cell death protein 1
JMOG: Japanese Musculoskeletal Oncology Group
FT1DM: Fulminant type 1 diabetes mellitus
